# Pioglitazone Ameliorates Mitochondrial Oxidative Stress and Inflammation via AMPK‐Dependent Inhibition of Mitochondrial Fission in Leigh Syndrome

**DOI:** 10.1111/cpr.70109

**Published:** 2025-08-06

**Authors:** Jie Luo, Ling Chen, Xiaoxian Zhang, Qiang Su, Xiaoya Zhou, Qizhou Lian

**Affiliations:** ^1^ Cord Blood Bank Centre, Guangzhou Women and Children's Medical Center Guangzhou Medical University Guangzhou China; ^2^ Department of Endocrinology, Shenzhen Second People's Hospital, Shenzhen Clinical Research Center for Metabolic Diseases Shenzhen Center for Diabetes Control and Prevention, the First Affiliated Hospital of Shenzhen University Shenzhen China; ^3^ CAS Key Laboratory of Quantitative Synthetic Biology, Shenzhen Institutes of Advanced Technology, Faculty of Synthetic Biology, Chinese Academy of Sciences Shenzhen University of Advanced Technology Shenzhen China; ^4^ Center for Translational Stem Cell Biology Hong Kong China; ^5^ HKUMed Laboratory of Cellular Therapeutics University of Hong Kong Hong Kong China; ^6^ Department of Surgery The University of Hong Kong Shenzhen Hospital Shenzhen China

## Abstract

Loss of function mutations of NDUFS4 resulted in Leigh syndrome, which is a progressive neurodegenerative disease and characterized by mitochondrial oxidative stress, inflammation and aberrant mitochondrial dynamics. However, there is currently no effective treatment. Here, we demonstrate that pioglitazone significantly mitigates mitochondrial reactive oxygen species (ROS) generation, lowers cyclooxygenase‐2 (COX‐2) mRNA levels, and rescues aberrant mitochondrial dynamics in vitro (increasing Opa‐1 expression while decreasing Drp‐1 expression). Furthermore, similar effects were observed with the selective Drp‐1 inhibitor mdivi‐1, suggesting that inhibiting mitochondrial fission mediates the therapeutic effects of pioglitazone. Pioglitazone administration activated AMPK phosphorylation, but these effects, along with pioglitazone's ability to reverse oxidative stress, inflammation, and mitochondrial fission, were abolished by the AMPK inhibitor compound C. In vivo, pioglitazone alleviated motor dysfunction, prolonged lifespan, and promoted weight gain in Ndufs4 KO mice. This was accompanied by enhanced mitochondrial fusion and increased levels of mitochondrial complex subunits. Consistently, pioglitazone attenuated neuroinflammation and oxidative stress in vivo. Collectively, our findings indicate that pioglitazone alleviates mitochondrial oxidative stress and inflammation through an AMPK‐dependent inhibition of Drp‐1‐mediated mitochondrial fission. Therefore, suppression of mitochondrial fission may represent a novel therapeutic strategy for Leigh syndrome (LS).

## Graphical abstract

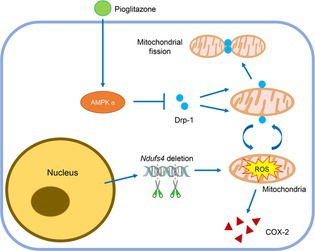

## 1 Introduction

Leigh syndrome (LS) is an inherited neurodegenerative disorder caused by mutations in over 75 genes encoding components of the mitochondrial respiratory chain (MRC). As the most common paediatric mitochondrial disease, it typically manifests in infancy, but unfortunately, most affected children succumb rapidly after onset [1, 2].

The MRC comprises five complexes (I‐V). Complex I (CI), the initial enzyme in the electron transport chain (ETC), generates electrons [3]. CI is assembled from dozens of subunits encoded by both nuclear and mitochondrial DNA. Dysfunction of CI can arise from various mutations, including those in Ndufs4, a gene critical for CI assembly and stability [4]. *Ndufs4* knockout (KO) models exhibit symptoms characteristic of LS, such as ataxia, muscle weakness, developmental delay, failure to thrive, and respiratory arrest [5, 6]. With an incidence of approximately 1 in 40,000 live births and a poor prognosis, LS remains a life‐threatening condition lacking effective treatments [6].

CI is a primary site for mitochondrial reactive oxygen species (ROS) generation [7], and its dysfunction is a major contributor to elevated mitochondrial ROS levels in LS [5]. Excessive mitochondrial ROS promotes pro‐inflammatory cytokine release by activating the NLRP3 inflammasome and triggering caspase activation during cellular damage [5, 8]. Inhibition of ROS using agents like 2R,4R‐aminopyrrolidine dicarboxylate or Mito‐TEMPO ameliorates inflammatory gene expression in *Ndufs4* KO neonates, highlighting the pivotal role of ROS in LS‐associated systemic inflammation [9].

Mitochondrial dynamics, involving the opposing processes of fusion and fission, is crucial for maintaining mitochondrial homeostasis [10]. Disruption of this balance leads to abnormal mitochondrial morphology, exacerbates oxidative stress and respiratory dysfunction, and ultimately contributes to neurodegeneration [11]. Mitochondrial fusion is regulated by the GTPases mitofusin 1/2 (Mfn1/2; outer membrane) and optic atrophy 1 (Opa‐1; inner membrane). Loss of either impairs fusion, causing mitochondrial fragmentation. Notably, Opa‐1 deficiency is also associated with disorganised mitochondrial cristae [9] and impaired assembly/activity of respiratory chain supercomplexes [12]. Overexpression of Opa‐1 in *Ndufs4* KO mice partially rescues mitochondrial function and extends lifespan, suggesting Opa1 as a potential therapeutic target for LS [9]. Conversely, excessive fission, mediated by dynamin‐related protein 1 (Drp‐1), typically promotes pathological mitochondrial fragmentation, impaired respiration, mitophagy, and apoptosis [11, 13]. Increased mitochondrial fission has been observed in fibroblasts from patients with Leigh Syndrome French Canadian type (LSFC) [14], though its role in LS neurons or glia remains unclear.

A bidirectional relationship exists between oxidative stress and mitochondrial dynamics [15, 16]. Oxidative stress predisposes neurons to mitochondrial fission prior to neuronal loss [17]. Conversely, modulating mitochondrial dynamics can mitigate excessive ROS production in neurodegeneration. For example, inhibiting Drp‐1 with the selective inhibitor P110 reduces fragmentation, ROS generation, and apoptosis in a Parkinson's disease model [18]. While this link remains unexplored in LS, it suggests that regulating mitochondrial dynamics could be a potential strategy for ROS scavenging and neuroprotection in LS.

Pioglitazone, a peroxisome proliferator‐activated receptor‐*γ* (PPAR‐*γ*) agonist commonly used to treat diabetes by improving insulin sensitivity, also demonstrates benefits beyond metabolic regulation. Recent studies indicate pioglitazone ameliorates mitochondrial dysfunction and suppresses inflammation in models of neurodegeneration, including Amyotrophic Lateral Sclerosis (ALS), Parkinson's disease, and adrenoleukodystrophy (ALD) [19, 20, 21]. Traditionally, mitochondrial biogenesis via PPAR‐*γ* coactivator‐1*α* (PGC‐1*α*) activation—inducing expression of NRF1, NRF2, and Tfam to enhance mitochondrial copy number and function—is considered a key pharmacological mechanism [22, 23]. Pioglitazone's anti‐inflammatory and antioxidant effects in neurological disorders are well documented [20, 21], though the precise mechanisms remain incompletely understood.

Pioglitazone can activate AMP‐activated protein kinase (AMPK) independently of PPAR‐*γ* in Swiss‐3 T3 cells [24], with similar effects reported in rat tissues [25], though its impact on neural or glial cells is unknown. Notably, C‐peptide prevents Drp‐1‐mediated mitochondrial fission in an AMPK‐dependent manner [26], underscoring AMPK's significant role in regulating fission.

The primary aim of this study was to investigate whether imbalanced mitochondrial dynamics promotes ROS overproduction and neuroinflammation in the pathogenesis of Leigh syndrome. A secondary aim was to determine whether pioglitazone administration ameliorates oxidative stress and inflammation by regulating mitochondrial dynamics in an AMPK‐dependent manner. Using primary mNPCs in vitro and *Ndufs4* KO mice in vivo, we designed experiments to address these questions.

## 2 Results

### 2.1 *Ndufs4* Knockout Predisposes mNPCs to Mitochondrial Dysfunction, ROS Generation, and Inflammation

**FIGURE 1:**
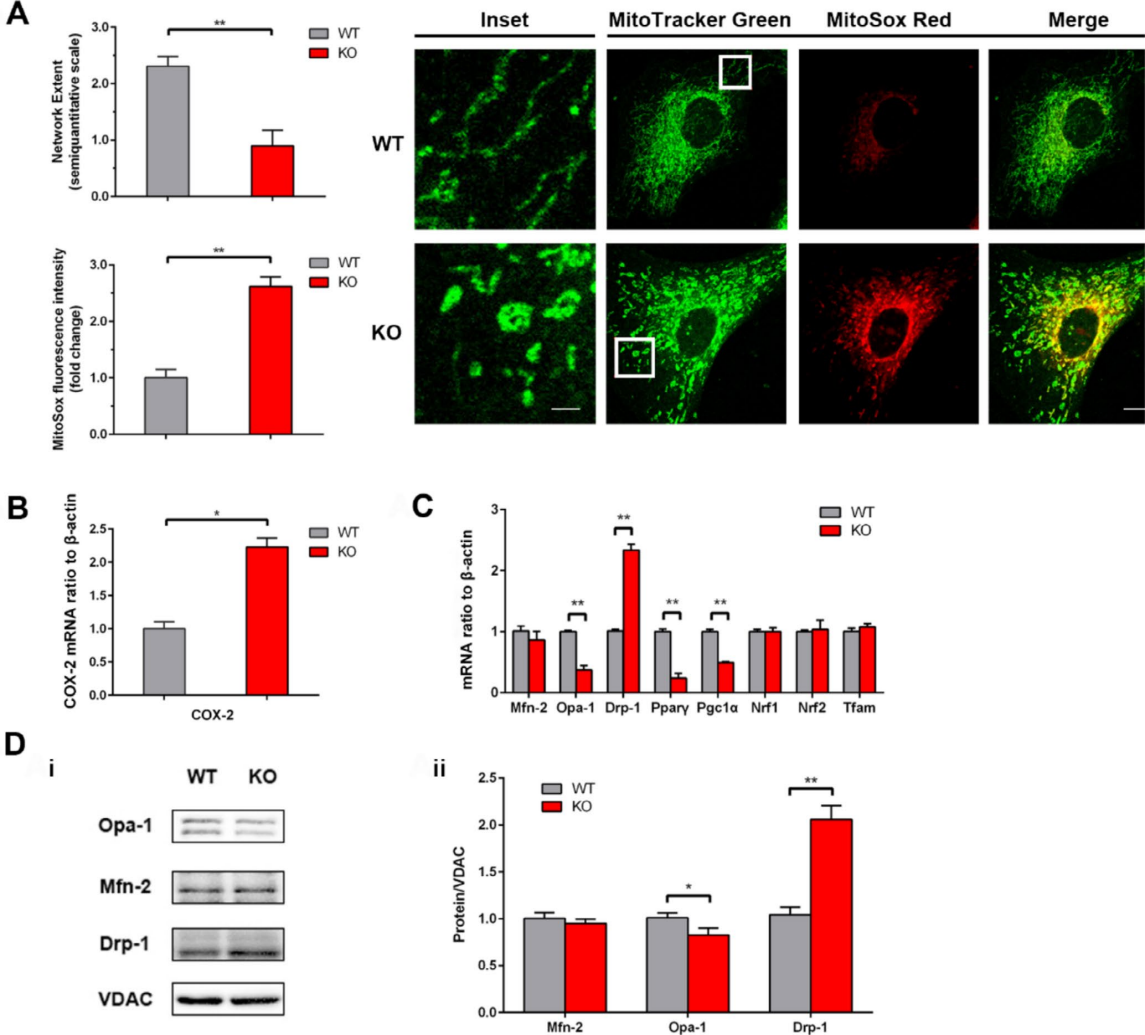
*Ndufs4* KO induces mitochondrial ROS generation, inflammation, and aberrant mitochondrial dynamics in mNPCs. (A) Measurement of mitochondrial morphology and ROS level labelled by MitoTracker Green and MitoSox Red. Scar bar = 10 μm; for inset, scar bar = 2 μm. (B) QPCR analysis of COX‐2 mRNA level relative to *Ndufs4* WT group. (C) QPCR analysis of the mRNA level of mitochondrial‐biogenesis‐related genes relative to *Ndufs4* WT group. (D) Western blot analysis of Mfn‐2, Opa‐1, and Drp‐1 relative to *Ndufs4* WT group. VDAC is taken as a loading control (i, ii). Data are presented as mean ± SEM of three independent experiments. *p < 0.05; **p < 0.01.

*Ndufs4* knockout (KO) in mNPCs led to mitochondrial ROS generation, inflammation, and aberrant mitochondrial dynamics. Compared to wild‐type (WT) mNPCs, which exhibited thread‐like and elongated mitochondria, primary cultured *Ndufs4* KO mNPCs displayed a fragmented mitochondrial network (Figure 1A). Quantitative analysis confirmed a significantly higher mitochondrial network extent score in WT mNPCs than in KO mNPCs (2.310 ± 0.175 vs. 0.900 ± 0.277; *p* < 0.01). Furthermore, ROS levels were markedly elevated in KO mNPCs relative to WT controls (2.619 ± 0.169 vs. 1.000 ± 0.149; *p* < 0.01; Figure 1A). These findings indicate that *Ndufs4* deficiency is associated with increased mitochondrial fission and oxidative stress. Given the established link between oxidative stress and inflammation, we assessed COX‐2 expression. mRNA analysis revealed significantly higher COX‐2 levels in KO mNPCs compared to WT (2.232 ± 0.134 vs. 1.000 ± 0.105; *p* < 0.05; Figure 1B), suggesting *Ndufs4* KO promotes an inflammatory state. Finally, we evaluated the expression of genes and proteins involved in mitochondrial biogenesis and dynamics. KO mNPCs exhibited aberrant mitochondrial dynamics characterised by increased Drp‐1 and decreased Opa‐1 expression at both the mRNA and protein levels compared to WT (Figure 1C,D). In contrast, Mfn2 expression remained unchanged. Similarly, mitochondrial biogenesis genes Nrf1, Nrf2, and Tfam showed no significant alterations, although Ppar*γ* and Pgc1*α* expression were significantly different (*p* < 0.01; Figure 1C).

### 2.2 Drp‐1 Inhibition by Mdivi‐1 Attenuates Mitochondrial ROS and Inflammation in *Ndufs4* KO mNPCs

**FIGURE 2:**
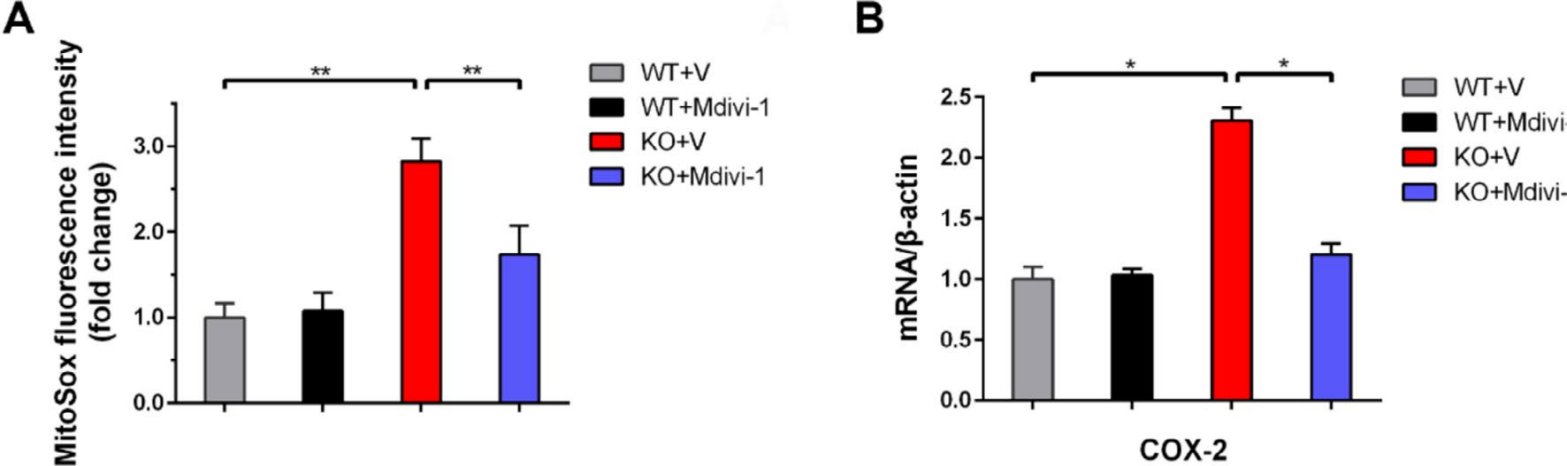
Mdivi‐1 inhibition of Drp‐1 expression reduces mitochondrial ROS and inflammation in mNPCs. (A) Mitochondrial ROS level relative to *Ndufs4* WT group 24 h after treatment. (B) QPCR analysis of COX‐2 mRNA level relative to *Ndufs4* WT group 24 h after treatment. Data are presented as mean ± SEM of three independent experiments. *p < 0.05; **p < 0.01.

Having confirmed downregulation of Opa‐1 and upregulation of Drp‐1 in *Ndufs4* KO mNPCs, and considering that Opa1 overexpression improves mitochondrial function in *Ndufs4* KO mice, we investigated whether specifically inhibiting Drp‐1‐mediated mitochondrial fission could rescue inflammation and mitochondrial oxidative stress. Treatment of *Ndufs4* KO mNPCs with Mdivi‐1, a specific Drp‐1 inhibitor, significantly suppressed mitochondrial ROS generation compared to vehicle‐treated controls (Figure 2A; *p* < 0.01). Additionally, Mdivi‐1 treatment significantly reduced COX‐2 expression in KO mNPCs versus the vehicle control (Figure 2B; *p* < 0.05). These results suggest that aberrant mitochondrial fission machinery in *Ndufs4* KO mNPCs drives excessive ROS production and inflammation.

### 2.3 Pioglitazone Mitigated Mitochondrial Dysfunction, ROS Generation, and Inflammation in *Ndufs4* KO mNPCs

**FIGURE 3:**
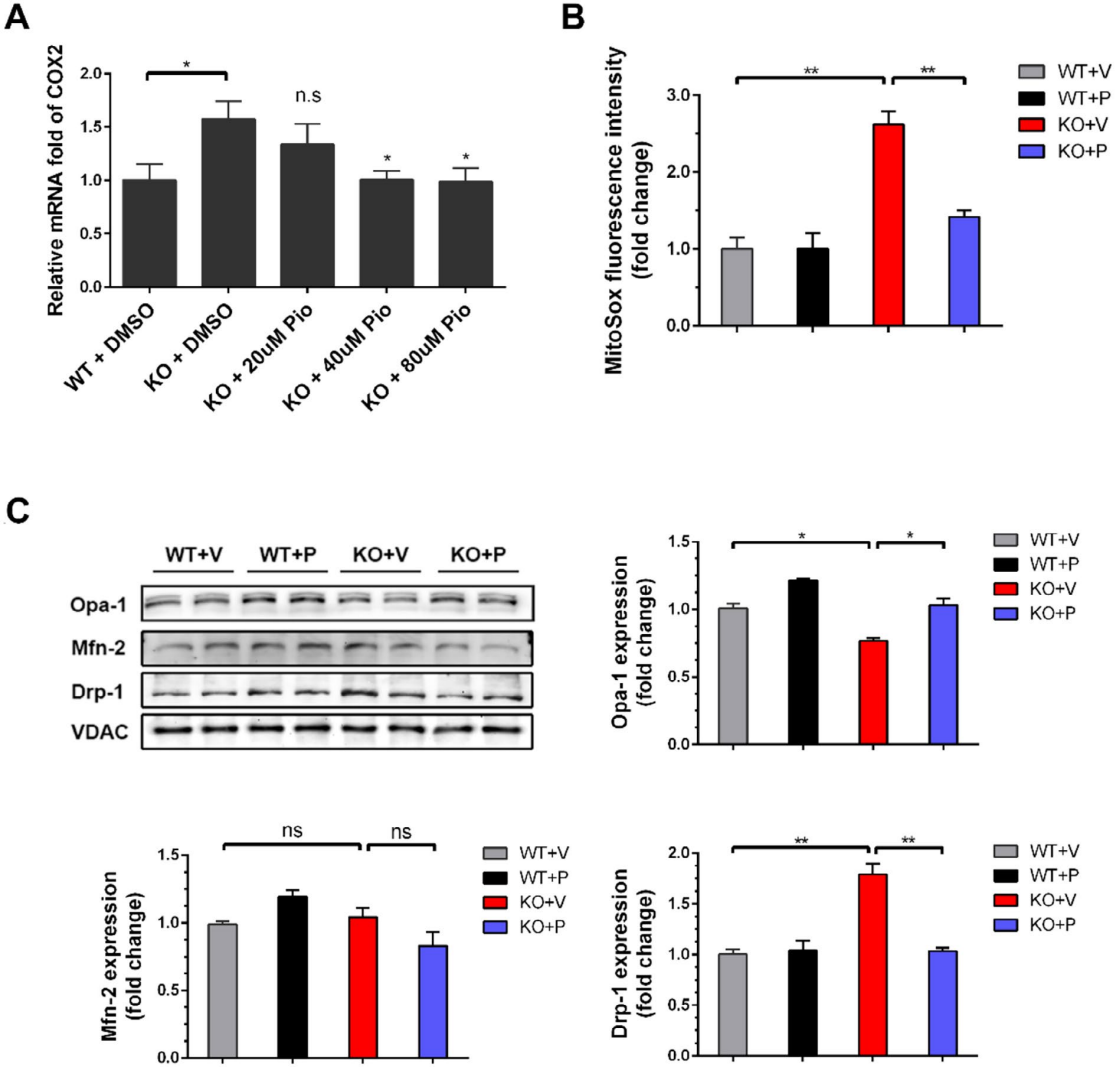
Pioglitazone attenuates mitochondrial ROS generation, inflammation, and ameliorates mitochondrial dynamics in mNPCs. (A) Dose‐dependent inhibition of COX‐2 mRNA level by pioglitazone (PIO) and DMSO relative to *Ndufs4* WT group 24 h after treatment. (B) Mitochondrial ROS level relative to *Ndufs4* WT group 24 h after treatment. (C) (i) Western blot of Opa‐1, Mfn‐2, Drp‐1, and VDAC 24 h after treatment. (ii–iv) Quantification represents the level of Opa‐1, Mfn‐2, and Drp‐1 normalised to VDAC relative to *Ndufs4* WT group. Data are presented as mean ± SEM of the three independent experiments. *p < 0.05; **p < 0.01; ns, not significant.

Given the demonstrated efficacy of pioglitazone in ameliorating mitochondrial dysfunction in various neurological disorders, we investigated its effects in *Ndufs4* KO mNPCs. As COX‐2 is a known target of pioglitazone [21], we confirmed its dose‐dependent inhibition in *Ndufs4* KO mNPCs, with optimal suppression observed at 40 μM after 24 h (Figure 3A). Correspondingly, pioglitazone significantly suppressed mitochondrial ROS generation (Figure 3B; *p* < 0.01). Western blot analysis of mitochondrial protein fractions revealed that pioglitazone significantly decreased Drp‐1 expression (*p* < 0.01) and increased Opa‐1 expression (*p* < 0.05) in *Ndufs4* KO mNPCs (Figure 3C), indicating enhanced mitochondrial fusion and suppressed mitochondrial fission. Consistent with the previous results, Mfn2 expression remained unchanged. Collectively, these findings demonstrate that pioglitazone ameliorates mitochondrial dysfunction, ROS generation, and inflammation in *Ndufs4* KO mNPCs.

### 2.4 AMPK Activation Mediates Pioglitazone's Rescue of Aberrant Mitochondrial Dynamics in *Ndufs4* KO mNPCs

**FIGURE 4:**
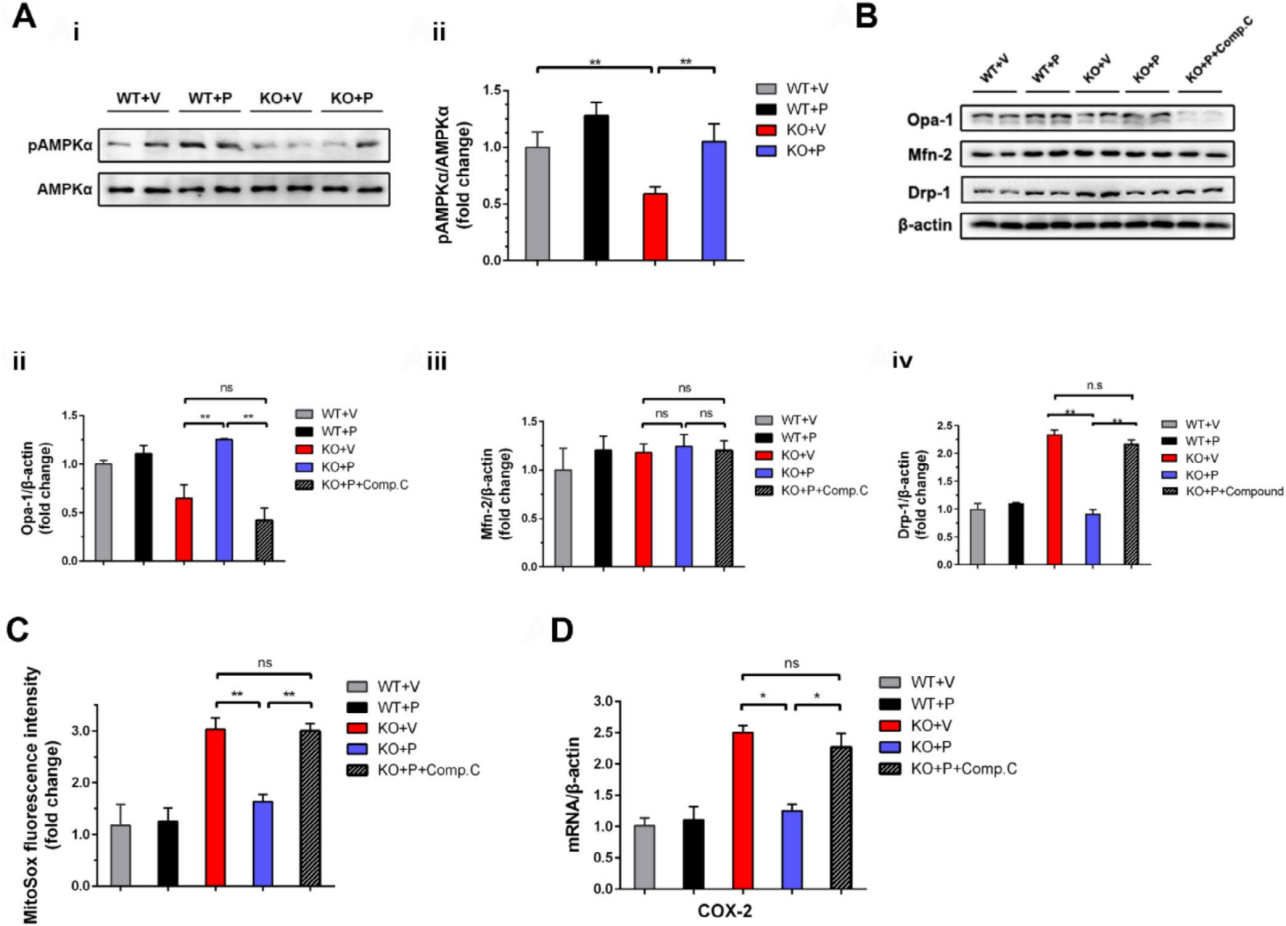
AMPK activation mediates pioglitazone's rescue of aberrant mitochondrial dynamics in *Ndufs4* KO mNPCs. (A) (i) Western blot of phospho‐AMPK*α* (Thr172) (pAMPK*α*) and AMPK*α* 24 h after treatment. (ii) Quantification represents the ratio of pAMPK*α*/AMPK*α* relative to *Ndufs4* WT group. (B) (i) Western blot of Opa‐1, Mfn‐2, and Drp‐1 24 h after treatment (compound C‐1 μM). (ii–iv) Quantification represents the levels of Opa‐1, Mfn‐2, and Drp‐1 normalised to *β*‐Actin. (C) Measurement of mitochondrial ROS relative to *Ndufs4* WT group 24 h after treatment. (D) QPCR analysis of COX‐2 mRNA level relative to *Ndufs4* WT group 24 h after treatment. Data are presented as mean ± SEM of three independent experiments. *p < 0.05; **p < 0.01; ns, not significant; Comp C, compound C.

While pioglitazone is known to activate AMPK, we investigated whether AMPK mediates its rescue of aberrant mitochondrial dynamics in *Ndufs4* KO mNPCs. Western blot analysis revealed significantly lower expression of phosphorylated AMPK*α* (p‐AMPK*α*) in *Ndufs4* KO mNPCs compared to WT mNPCs (*p* < 0.01), with levels approximately half those of WT cells (Figure 4A). Notably, pioglitazone treatment significantly normalised p‐AMPK*α* levels in KO mNPCs relative to vehicle‐treated controls (*p* < 0.01), confirming AMPK*α* pathway activation in this model.

To determine if pioglitazone ameliorates mitochondrial dysfunction via AMPK*α* regulation, we used the AMPK inhibitor compound C. Compared to pioglitazone treatment alone, co‐treatment with pioglitazone and compound C in KO mNPCs resulted in a significant decrease in Opa‐1 and a concomitant increase in Drp‐1 (Figure 4B; *p* < 0.01). Compound C alone had no significant effect on Mfn‐2 (Figure 4B; *p* > 0.05). Importantly, the expression levels of Opa‐1 and Mfn‐2 in the pioglitazone‐plus‐compound C group were not significantly different from those in the vehicle‐treated group (Figure 4B; *p* > 0.05).

Similarly, compound C partially reversed the effects of pioglitazone on mitochondrial ROS production and COX‐2 mRNA levels. Both mitochondrial ROS fluorescent intensity and COX‐2 mRNA expression were significantly higher in KO mNPCs treated with pioglitazone plus compound C than in those treated with pioglitazone alone (Figure 4B–D; *p* < 0.01 and *p* < 0.05, respectively). However, levels in the combination group did not differ significantly from the vehicle‐treated group (*p* > 0.05). These findings indicate that pioglitazone modulates mitochondrial dynamics primarily through AMPK signalling.

### 2.5 Pioglitazone Improved Motor Function and Extended the Lifespan of *Ndufs4* KO Mice

**FIGURE 5:**
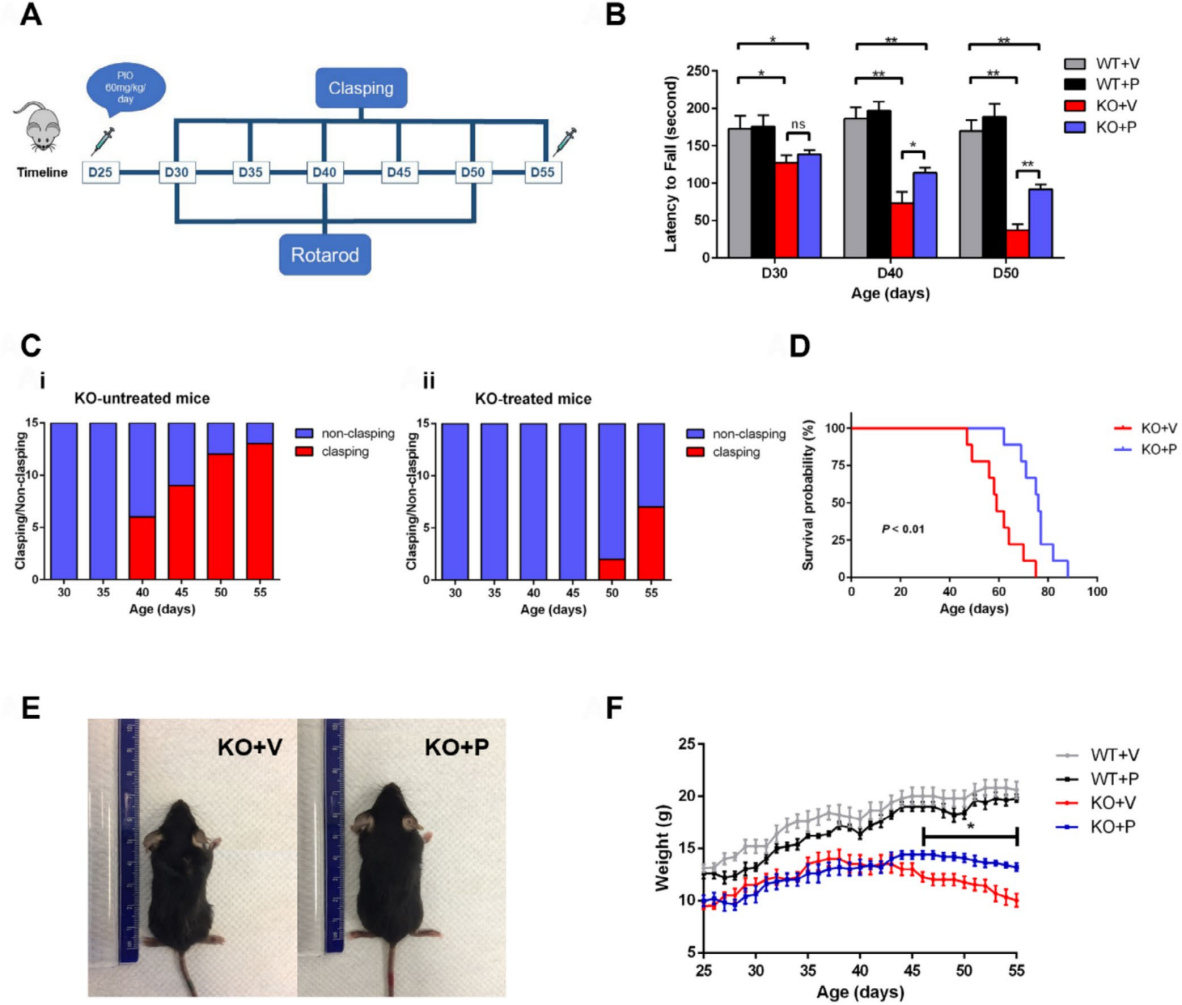
Pioglitazone improves motor function and prolongs lifespan of *Ndufs4* KO mice. (A) Schematic chart showing the timeline of animal neurobehavioral tests during D25 to D55. (B) Rotarod analysis for different groups at D30, D40, and D50 (*n* ≥ 6 for each group). (C) Ratio of non‐clasing/clasing between vehicle‐treated (i) and pioglitazone‐treated (ii) *Ndufs4* KO mice (*n* = 15 for each group). (D) Kaplan–Meier survival curve (*n* = 9 for each group). Significance is assessed by the log rank test. (E) Comparison of body size between vehicle‐treated (KO + V) and pioglitazone‐treated (KO + P) *Ndufs4* KO mice at D55. (F) Body weight monitoring of different groups of mice. Data are presented as mean ± SEM. *p < 0.05; **p < 0.01; ns, not significant.

To further validate pioglitazone's therapeutic efficacy, we conducted experiments in *Ndufs4* KO mice using the timeline and protocol outlined (Figure 5A). Gross motor function and neurodegeneration were assessed via rotarod testing and clasping behaviour. The rotarod test revealed that pioglitazone significantly delayed neurological decline (Figure 5B). Compared to vehicle‐treated KO mice, pioglitazone‐treated KO mice exhibited improved performance at D40 (*p* < 0.05) and D50 (*p* < 0.01). Despite treatment, KO mice consistently scored lower than WT mice across time points (*p* < 0.01 and *p* < 0.05, respectively). Additionally, treated mice displayed clasping—a sign of neurodegeneration—less frequently than untreated controls after D40 (Figure 5C).

Kaplan–Meier analysis confirmed that pioglitazone prolonged the median lifespan of KO mice (76.0 vs. 59.0 days; log‐rank test, *p* < 0.01; Figure 5D). Weight analysis showed that pioglitazone‐treated KO mice gained more weight than untreated counterparts, becoming significantly heavier by D46 (*p* < 0.05), though both groups exhibited progressive weight loss in later stages (Figure 5F). At D55, treated KO mice also displayed larger body size. In contrast, WT mice weight remained largely unaffected (Figure 5E). These results suggested that pioglitazone is an effective treatment for the LS model.

### 2.6 Pioglitazone Ameliorated Central Nervous System (CNS) Inflammation in *Ndufs4* KO Mice

**FIGURE 6:**
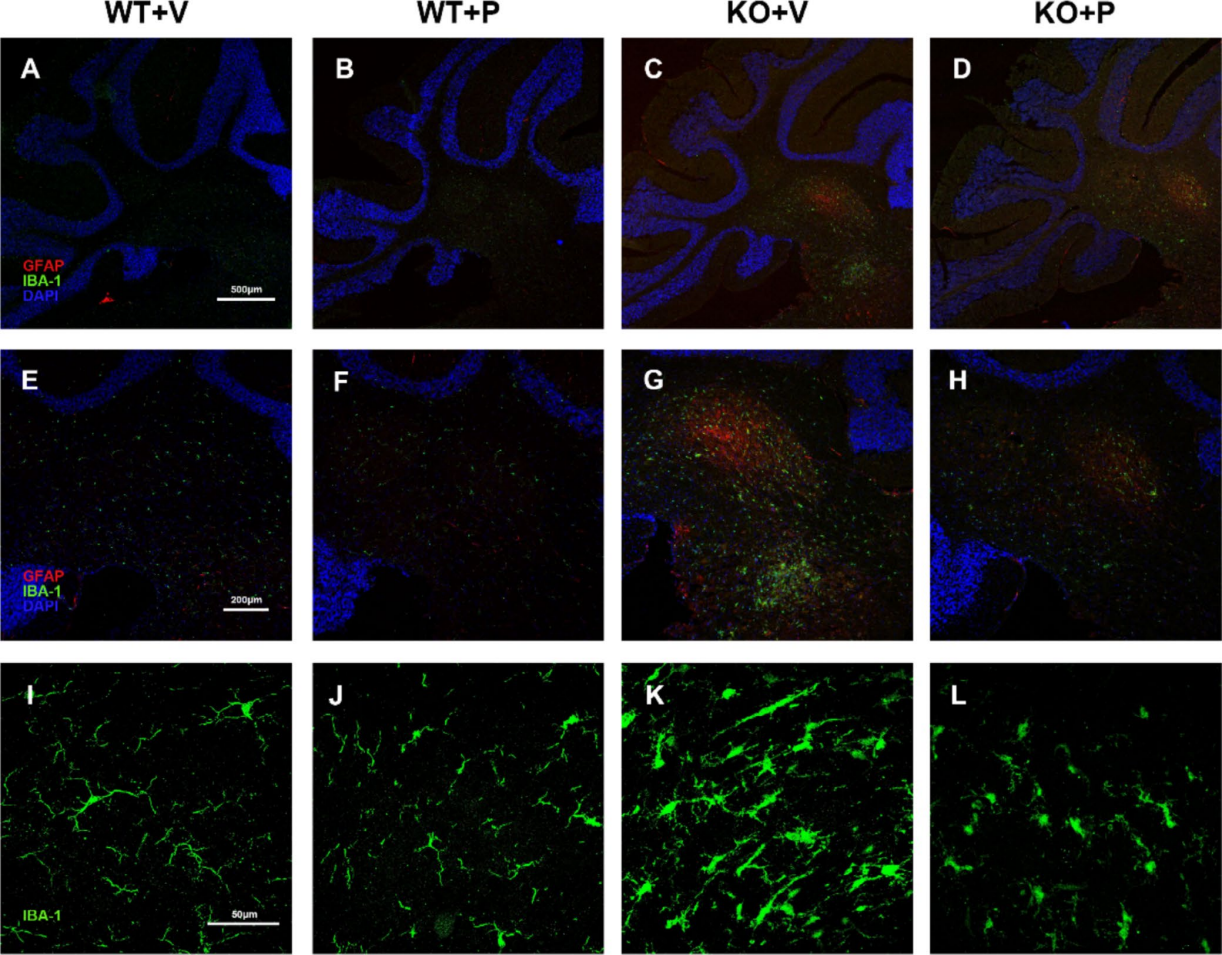
Pioglitazone alleviates neuroinflammation in the cerebellum of *Ndufs4* KO mice. (A–D) Representative immunostaining for glia activation by Iba‐1 and GFAP in the cerebellum at D55 among different groups (*n* = 6 for each group). Scar bar = 500 μm. (E–H) High magnification of local area in the cerebellum. Scar bar = 200 μm. (I–L) Morphological change of microglia in the cerebellum. Scar bar = 50 μm.

Building upon in vitro evidence that pioglitazone reduces mRNA expression of the inflammatory mediator COX‐2, we further assessed its anti‐inflammatory effects in vivo. By the experimental endpoint (D55), vehicle‐treated *Ndufs4* KO mice developed severe CNS inflammation, characterised by robust glial activation (detected by GFAP and Iba‐1 immunostaining; Figure 6C). These inflammatory lesions were predominantly localised to the deep cerebellar nuclei, with minimal involvement of the cerebellar lobes (Figure 6G). High‐magnification imaging revealed intensely activated microglia within these nuclear regions of KO mice, exhibiting enlarged cell bodies, linear morphologies, and dense clustering (Figure 6K). In contrast, microglia in wild‐type (WT) mice maintained a resting state (Figure 6A,E,I).

Daily administration of pioglitazone significantly attenuated neuroinflammation within the cerebellar nuclei of *Ndufs4* KO mice compared to vehicle‐treated controls (Figure 6D). Under high‐power magnification, pioglitazone suppressed glial activation and ameliorated the activated morphology of microglia (Figure 6H,L). Furthermore, pioglitazone treatment did not alter Iba‐1 or GFAP expression or distribution in WT mice (Figure 6B,F), and resting microglia were consistently observed in the cerebellum of WT mice regardless of treatment (Figure 6I,J). The above experimental results demonstrated that pioglitazone exerts a central anti‐inflammatory effect in the LS model.

### 2.7 Pioglitazone Promoted Mitochondrial Fusion and Improved Mitochondrial Function in *Ndufs4* KO Mice

**FIGURE 7:**
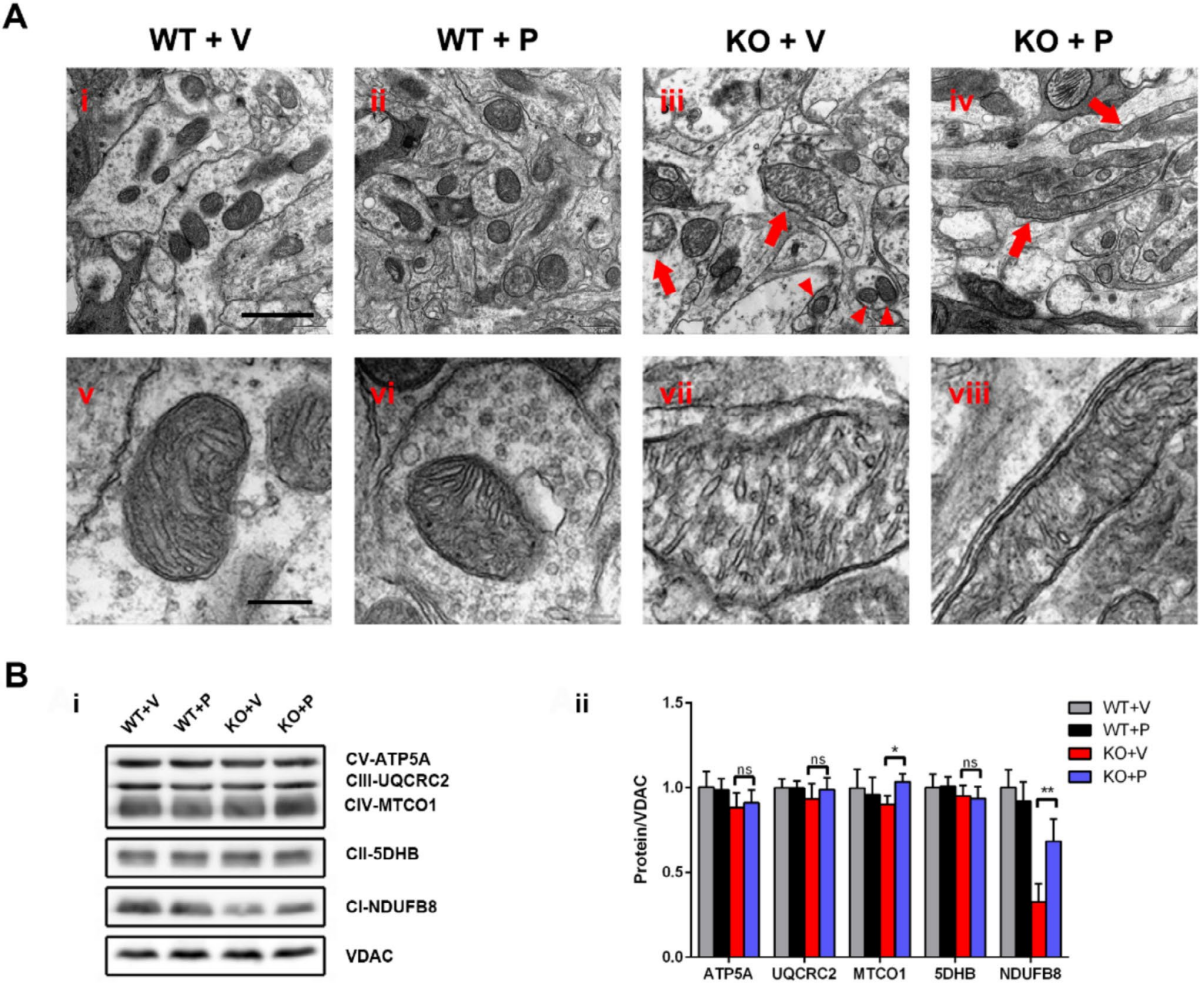
Pioglitazone enhances the mitochondrial fusion and increases MRC subunits expression. (A) EM of mitochondrial ultrastructure in mice brain (D55) among different groups at lower (i–iv) and higher (v–viii) magnification (*n* = 4 for each group). Scale bar = 500 nm; scale bar of the inset = 100 nm. (B) Western blot analysis of MRC subunits expression relative to *Ndufs4* WT group. VDAC is taken as a loading control. Data are presented as mean ± SEM of three independent experiments. *p < 0.05; **p < 0.01; ns, not significant.

We further assessed the drug's effect on mitochondrial ultrastructure in vivo. Electron microscopy (EM) analysis of brain tissue from KO mice revealed significant ultrastructural abnormalities, including vacuolar degeneration within the inner mitochondrial compartment and disorganised cristae (Figure 7Aiii, arrow; and 7Aviii). Additionally, a subset of mitochondria appeared dramatically fragmented (Figure 7Aiii, arrowhead). In contrast, mitochondria in pioglitazone‐treated KO mice exhibited remarkable fusion (Figure 7Aiv,viii, arrow), with concomitant amelioration of cristae disorganisation and vacuolar degeneration.

Mitochondrial structure remained intact and normal in WT mice regardless of vehicle or pioglitazone treatment (Figure 7Ai,ii,v,vi). Similarly, both vehicle‐ and pioglitazone‐treated WT mice displayed normal mitochondrial morphology with clear and ordered cristae. In contrast, mitochondrial fission was prevalent in both vehicle‐ and pioglitazone‐treated KO groups. Notably, pioglitazone treatment did not improve mitochondrial structure in the skeletal muscle of *Ndufs4* KO mice (data not shown).

Given that mitochondrial function is intimately linked to structure, we investigated potential functional changes. Western blot analysis of isolated brain mitochondria demonstrated that pioglitazone treatment significantly enhanced the expression of two mitochondrial respiratory chain (MRC) subunits, NDUFB8 (Complex I) and MTCO1 (Complex IV), compared to vehicle‐treated KO mice (Figure 7B; *p* < 0.01 and *p* < 0.05, respectively, Student's *t*‐test). However, no significant changes were observed in the expression levels of ATP5A (Complex V), UQCRC2 (Complex III), or SDHB (Complex II) between the two KO groups (Figure 7B; *p* > 0.05).

### 2.8 Pioglitazone Activates AMPK*α* to Inhibit Mitochondrial Fission, ROS Generation, and Inflammation in *Ndufs4* KO Mice

**FIGURE 8:**
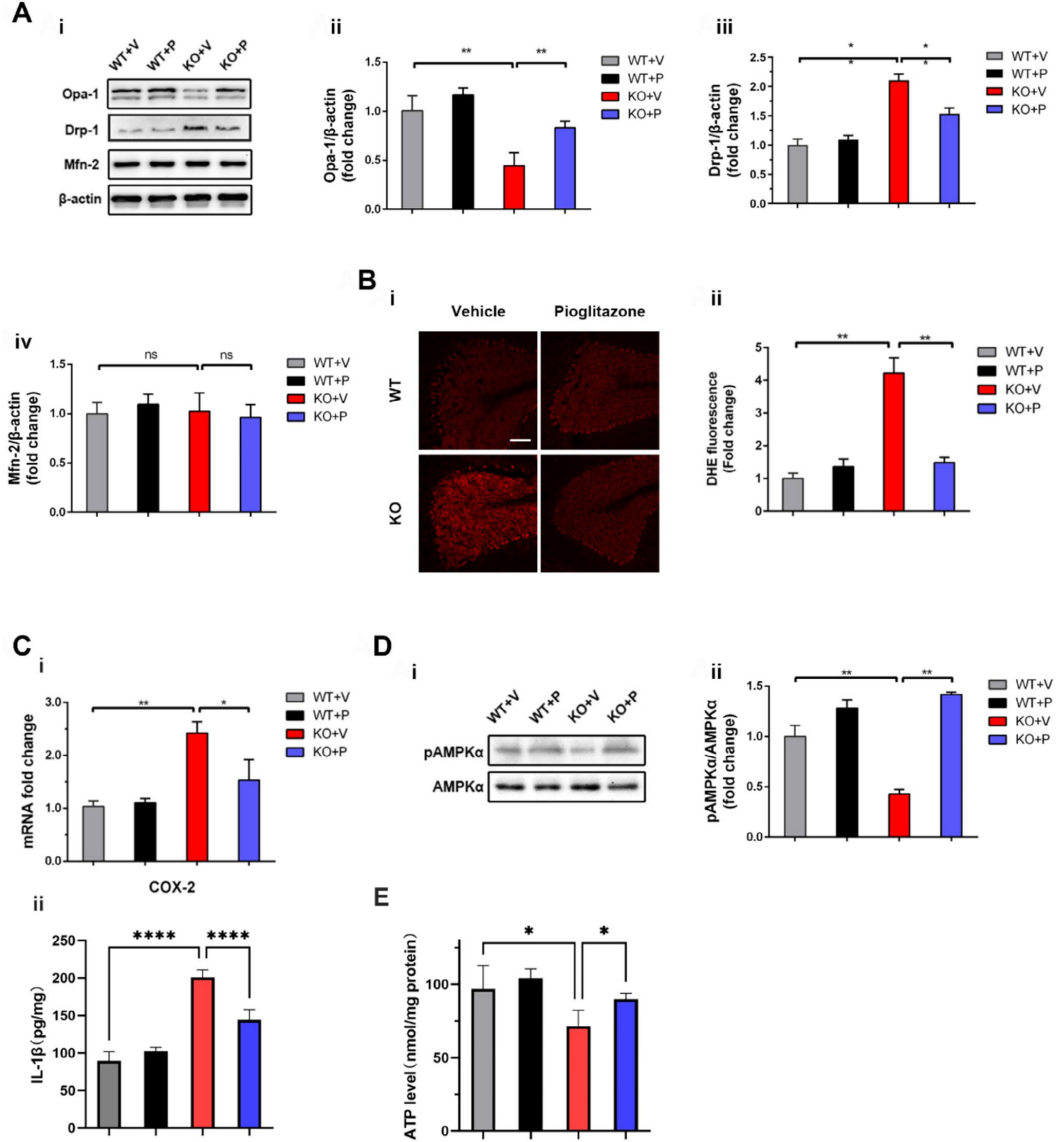
Pioglitazone‐induced AMPK activation inhibits mitochondrial fission, ROS generation and inflammation in *Ndufs4* KO mice. (A) Representative immunoblot and quantitative analysis of mitochondrial dynamics related proteins of Opa‐1, Drp‐1 and Mfn‐2 (*n* = 6 for each group) (i–iv). (B) Representative images and quantification of ROS generation relative to *Ndufs4* WT group measured by DHE staining in cerebellum lobes (*n* ≥ 5 for each group). (C) (i) QPCR analysis of COX‐2 mRNA level relative to *Ndufs4* WT group (*n* = 6 for each group). (ii) ELISA was performed to measure the inflammatory cytokine IL‐1*β* in brain tissue (*n* ≥ 3). (D) Representative immunoblot and quantitative analysis of pAMPK*α* expression. Quantification of pAMPK*α* was normalised to AMPK*α* (*n* = 6 for each group). Data are presented as mean ± SEM. *p < 0.05; **p < 0.01; ns, not significant. E. ATP detection in brain tissue (*n* ≥ 3).

At the molecular level, compared to vehicle‐treated KO mice, pioglitazone‐treated KO mice exhibited upregulated Opa‐1 expression and downregulated Drp‐1 expression (Figure 8A; *p* < 0.01), while Mfn‐2 levels remained unchanged (*p* > 0.05). Consistent with its role as a core product of mitochondrial metabolism, ATP content was significantly reduced in the brains of *Ndufs4* KO mice. Pioglitazone treatment restored this ATP depletion (Figure 8E), aligning with previous findings. Correspondingly, pioglitazone significantly attenuated mitochondrial ROS generation in these mice (Figure 8B; *p* < 0.01). Furthermore, pioglitazone decreased COX‐2 mRNA expression in KO mice, replicating prior in vitro observations. Additionally, pioglitazone treatment significantly reduced cerebellar IL‐1*β* levels (Figure 8Ci,ii), further confirming its broad anti‐inflammatory efficacy. An elevation in phosphorylated AMPK*α* (p‐AMPK*α*) protein levels was also detected in pioglitazone‐treated KO mice (Figure 8D). Collectively, these results suggest that pioglitazone mitigates mitochondrial fission, ROS generation, and inflammation in *Ndufs4* KO mice via AMPK*α* activation.

## 3 Discussion

In this study, we demonstrate that genetic deletion of the mitochondrial complex I subunit *Ndufs4* triggers excessive mitochondrial reactive oxygen species (mtROS) generation and elevated COX‐2 expression, concomitant with aberrant mitochondrial dynamics. Critically, pioglitazone administration rescued this dysfunctional state, effectively normalising the imbalance between mitochondrial fusion and fission. This normalisation was achieved through pioglitazone's dual action: enhancing the expression of the fusion protein Opa‐1 and suppressing the expression of the fission protein Drp‐1.

Our data strongly support a model where pioglitazone exerts its antioxidant and anti‐inflammatory effects in the Leigh syndrome (LS) model primarily through the inhibition of Drp‐1‐mediated mitochondrial fission, and this process is dependent on AMP‐activated protein kinase (AMPK) activation. This conclusion is underpinned by the finding that both Mdivi‐1 (a specific Drp‐1 inhibitor) and compound C (an AMPK inhibitor) completely abolished the beneficial effects of pioglitazone on mtROS, inflammation, and fission dynamics. The essential role of AMPK activation in mediating pioglitazone's suppression of Drp‐1 and subsequent mitochondrial protection is thus clearly established.

Mitochondrial dynamics, involving constant fusion and fission [27, 28], are fundamental to cellular health and are intricately linked to processes like mtROS generation, stress responses, and cell fate [29]. Complex I deficiency in LS is characterised by significant oxidative stress and inflammation [30], with mtROS acting as a key trigger for inflammation and apoptosis [31, 32]. Importantly, a bidirectional relationship exists between mtROS and mitochondrial dynamics: excessive ROS promotes fission [16, 26], and our findings demonstrate that inhibition of fission (via pioglitazone or directly with mdivi‐1) effectively mitigates mtROS and inflammation. This suggests a potential regulatory loop where fission and ROS mutually reinforce each other. Our results align with studies in other neurodegenerative diseases (e.g., PD [18], HD [33]), where inhibiting excessive mitochondrial fission (e.g., using P110 inhibitors) similarly ameliorates ROS overproduction, mitochondrial fragmentation, and dysfunction, reinforcing the therapeutic potential of targeting fission dynamics.

The balance between mitochondrial fusion and fission is critical, akin to a seesaw where attenuation of one process often enhances the other [34]. Pioglitazone uniquely modulated this balance bilaterally by upregulating Opa‐1 (fusion) and downregulating Drp‐1 (fission). This synchronous action is significant, as mitochondrial fragmentation in pathologies often results from both increased fission and reduced fusion [35, 36, 37]. While Opa‐1 upregulation is known to improve mitochondrial function in *Ndufs4* KO mice by enhancing cristae organisation, respiratory supercomplex stability, and respiratory efficiency [12], our study specifically highlights the crucial contribution of “Drp‐1 inhibition” in attenuating mtROS and inflammation. Suppressing fission may further promote fusion [38], potentially enabling defective mitochondria to complement each other through content exchange, maximising their functional capacity against stressors like ROS [39]. This mechanism likely underpins the benefits observed from inhibiting Drp‐1‐mediated fission.

AMPK, a central cellular energy sensor [40], was pharmacologically activated by pioglitazone in our model. While the precise mechanism of pioglitazone‐induced AMPK activation in this context warrants further investigation, evidence suggests PPAR‐*γ*‐independent pathways exist. These may involve TZD‐induced adiponectin release activating AMPK or AMP accumulation secondary to transient complex I inhibition [24]. The latter appears somewhat paradoxical given our observation of increased complex I subunit expression (e.g., NDUFB8), potentially indicating compensatory upregulation or fusion‐mediated rescue, necessitating future clarification regarding complex I activity. Crucially, regardless of the initiating mechanism, our data confirm that AMPK activation is functionally required for pioglitazone to inhibit Drp‐1, reduce fission, and alleviate oxidative stress/inflammation in *Ndufs4*‐deficient cells.

The role of AMPK in regulating Drp‐1 and mitochondrial fission is complex and context‐dependent. While AMPK deletion promotes fragmentation, Drp‐1 is recognised as a downstream target of AMPK in various tissues (e.g., inhibiting fission in diabetic adipose, hyperglycemic endothelium, *β*‐cells [38, 41, 42]); other studies report AMPK activation promoting fission and mitophagy under specific stress conditions by phosphorylating MFF and recruiting Drp‐1 [43]. Our results in the specific context of complex I deficiency and pioglitazone treatment unequivocally demonstrate that AMPK activation leads to the inhibition of Drp‐1‐mediated fission and confers protection. This underscores the importance of cellular context and the nature of the stressor in determining AMPK's downstream effects on mitochondrial morphology.

Beyond the cellular mechanism, daily pioglitazone administration in *Ndufs4* KO mice yielded significant therapeutic benefits: improved motor function, delayed disease onset, and extended lifespan. RNA sequencing of the cerebral cortex revealed that pioglitazone treatment shifted the gene expression profile of KO mice towards that of wild‐type mice, rescuing impairments in learning, memory, and cognition (Figure S1). The amelioration of neuroinflammation and oxidative stress within the CNS is central to this phenotypic rescue. Given that glia‐driven inflammation contributes to neurodegeneration and neuronal loss [44], its attenuation likely explains, at least in part, the recovery of motor neuron function and the delayed onset of neurodegenerative symptoms (e.g., clasping). Furthermore, since mitochondrial morphology in skeletal muscle remained unchanged, the improvement in overall motor function is most likely attributable to lesion reversal within the CNS, rather than in peripheral extremities. Notably, the lack of mitochondrial morphological changes in skeletal muscle suggests the motor improvements are primarily CNS‐mediated. Pioglitazone's benefits in neurodegeneration likely involve multiple pathways [20, 21]. While enhancing mitochondrial biogenesis via PGC‐1*α*is a recognised mechanism [22], our study highlights the crucial role of modulating dynamics. Interestingly, crosstalk exists, as PGC‐1*α* overexpression can suppress Drp‐1‐mediated fission [45, 46]. Given that PPAR‐*γ* (pioglitazone's primary target) is co‐activated by PGC‐1*α*, a joint regulatory role of AMPK and PGC‐1*α* in suppressing pathogenic fission via Drp‐1 is a plausible mechanism requiring future exploration.

Several limitations of the current study should be acknowledged. Firstly, while primary cultured mouse neural progenitor cells (mNPCs) capture key aspects of LS, primary neurons or microglia might offer a more physiologically relevant model for specific mechanistic studies. Secondly, although mdivi‐1 and compound C are widely used as specific inhibitors for Drp‐1 and AMPK, respectively, genetic approaches (e.g., siRNA, CRISPR/Cas9 knockdown/knockout) would provide more definitive validation of target involvement. Complementary studies using AMPK activators (e.g., AICAR, metformin) or genetic AMPK activation in vivo are warranted. Thirdly, potential side effects of pioglitazone (e.g., weight gain, fluid retention, bone loss) were not monitored during the in vivo experiments. Establishing an optimal dose balancing efficacy and tolerability is crucial for potential translation. Finally, the association of long‐term pioglitazone use with increased bladder cancer risk remains a clinical concern, potentially necessitating structural modifications or careful patient selection in future therapeutic applications.

## 4 Conclusion

**FIGURE 9:**
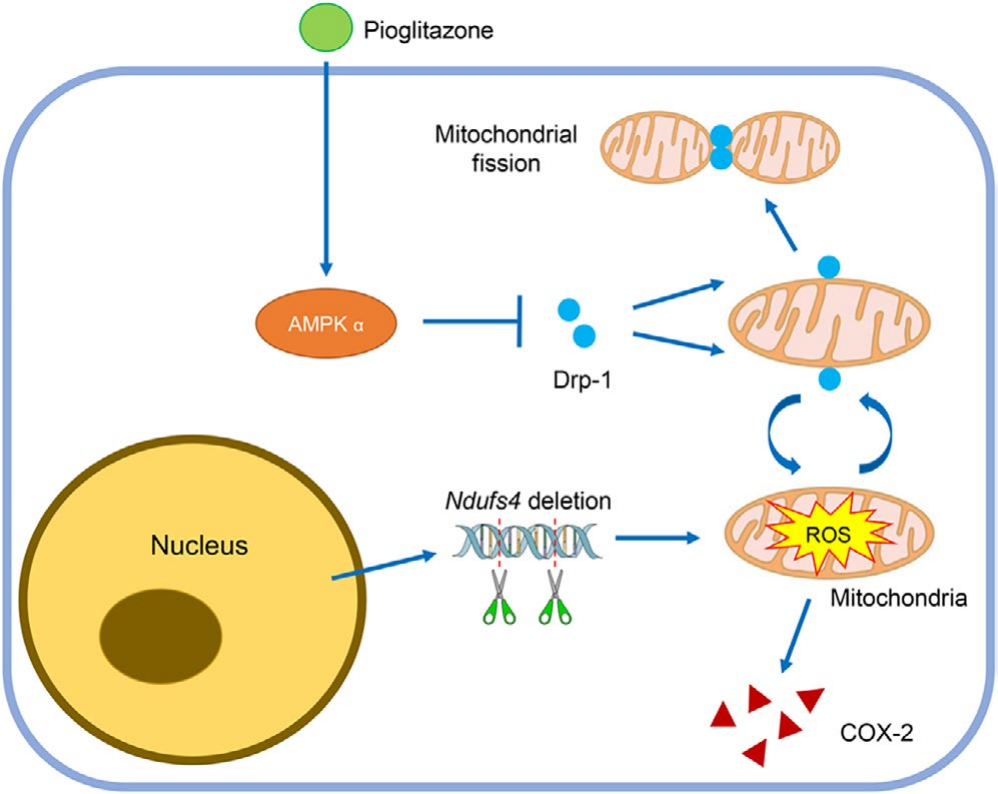
Schematic of the proposed mechanism that pioglitazone inhibits mitochondrial ROS and inflammation in LS. Pioglitazone suppresses mitochondrial excessive ROS generation and COX‐2 level through inhibition of Drp‐1 mediated mitochondrial fission in an AMPK‐dependent manner in LS.

In conclusion, current research demonstrates that genetic deletion of mitochondrial respiratory CI subunit *Ndufs4* leads to mitochondrial oxidative stress, inflammation, and aberrant mitochondrial dynamics. Pioglitazone normalises the imbalance between mitochondrial fusion and fission. In addition to the role of Opa‐1 in the rescue of mitochondrial dysfunction, research supports that pioglitazone suppresses mitochondrial oxidative stress and inflammation through inhibition of Drp‐1 mediated mitochondrial fission in an AMPK‐dependent manner in LS (Figure 9). It also indicates that pioglitazone improves motor function, delays disease progression, and prolongs the lifespan of LS. These findings strongly suggest that pioglitazone inhibition of mitochondrial fission may be a therapeutic strategy for preventing and treating LS.

## 5 Materials and Methods

### 5.1 Cell Culture

Mice neural precursor cells (mNPCs) were isolated from E12.5 *Ndufs4* mouse embryo forebrain cortex as previously described [47]. The genotyping procedure was done after the isolation. Cells were cultured in DMEM/F12 culture media supplemented with 2% B27, 100 units/mL penicillin/100 μg/mL streptomycin, 20 ng/mL Epidermal Growth Factor, and 10 ng/mL fibroblast growth factor. Passages between 5 and 8 were used. Cells were treated with 10 μM Mdivi for 24 h during culture. In functional assays, cells were treated with 10 μM Mdivi‐1 for 24 h [48].

### 5.2 Mitochondrial Staining and Analysis

Mitochondrial ROS generation and morphology were stained using MitoSox Red and MitoTracker Green (Invitrogen). Cells were seeded on the gelatin‐coated well and incubated with 1.25 μM MitoSox Red and 200 nM MitoTracker Green in PBS solution at 37°C for 15 min. ROS was verified within the mitochondria when both red and green signals were colocalised, and its fluorescence intensity was calculated with fold change in *Ndufs4* KO versus WT mNPCs. Mitochondrial fusion and fission were scaled by the network extent as described previously [49].

### 5.3 Animal Studies

Animals were housed under controlled conditions with a 12‐h light/dark cycle and received normal chow and water ad libitum. *Ndufs4* wildtype (WT) and knockout (KO) mice were grouped into four groups: WT + V (vehicle), WT + *P* (pioglitazone), KO + V, and KO + *P*. All mice were treated with an equivalent volume (15 μL/g) of vehicle or pioglitazone via daily intraperitoneal injection (I.P.) since day 25. Pulverised pioglitazone (Actos from Takeda, 15 mg/tablet) 4.0 mg was dissolved in 1 mL mixed solution (30ul EtOH, 50ul PEG‐400, 1.2ul HCL and 918.8ul 0.9% saline). Dosage of pioglitazone was 60 mg/kg/day. Measurement of weight and clasping were recorded since D25 and D35 with an interval of 1 and 5 days, respectively.

### 5.4 Rotarod

Rotarod instrument was used to evaluate mice motor function alteration at D30, D40, and D50 with two consecutive sessions for training and one session for testing at an accelerated speed of 0.1 rpm/s from resting status. Three to five replicates of each test were averaged.

### 5.5 Immunofluorescence

Mice were sacrificed at D55 under anaesthesia and perfused with PBS solution. Brain tissues were harvested and fixed in 4% PFA overnight, followed by sequential dehydration in 10%, 20% and 30% sucrose for 24 h, respectively, until being embedded in OCT. 30 μm frozen sections were used. Sections were blocked with 5% bull serum albumin (BSA) in PBS/0.5% Triton‐100 for 1 h at room temperature and incubated overnight at 4°C in a wet chamber with primary antibodies diluted in 5% BSA‐PBS. Primary antibodies are anti‐Iba1 (1:200, #019‐19741 from WAKO) and anti‐GFAP (1:400, #G3893) from Sigma. After being washed in PBS, slides were incubated for 2 h at room temperature with Goat anti‐Rabbit IgG (H + L) Cross‐Adsorbed Secondary Antibody, Alexa Fluor 488 (#A‐11008) and F(ab′)2‐Goat anti‐Mouse IgG (H + L) Cross‐Adsorbed Secondary Antibody, Alexa Fluor 568 (#A‐11019) from ThermoFisher Scientific. Sections were washed in PBS and counterstained with DAPI, and then coverslipped with gold antifade reagent (#P36930 from ThermoFisher Scientific). Images were taken with Carl Zeiss LSM 700 confocal fluorescence microscope.

### 5.6 Electron Microscope

Cerebellum and soleus were dissected and fixed in 2.5% glutaraldehyde in cacodylate buffer (0.1 M sodium cacodylate‐HCl buffer pH 7.4) overnight at 4°C, which was replaced by 0.1 M sucrose to stop fixation until being embedded in epoxy resin for the subsequent procedure for EM. Sections were examined with Philips CM100 TEM.

### 5.7 Tissue ROS Staining

ROS of brain tissue were detected by dihydroethidium (DHE) probe. Brain frozen sections were incubated in DHE (5 μM) at room temperature for 30 min. Following PBS wash, stained tissues were mounted with coverslip and proceeded to be observed by the confocal microscope.

### 5.8 Western Blot

Mitochondrial fraction in mouse tissues and cells was performed either as described in Gabriele et al. [4] or by using the Cytosol/Mitochondrial Fractionation Kit (Calbiochem) based on the manufacturer's instructions. Protein concentration was determined by the BCA protein assay kit (BosterBio) according to the instructions. Samples were heated at 37°C (only for OXPHOS subunits detection) or 100°C (for other proteins) for 10 min, loaded in 15–20 μg per well and run on SDS/PAGE. Transferred membranes were blocked with 5% milk‐TBST for 1 h and incubated with primary antibodies overnight at 4°C, including the OXPHOS antibody cocktail (NDUFB8, SDHB, UQCRC2, MTCO1 and ATP5A; 6ug/ml, #ab110413 from Abcam), anti‐Mfn2 (1: 1000, #sc‐50331 from Santa Cruz), anti‐Drp1 (1: 1000, #sc‐32898 from Santa Cruz; 1:1000, #ab184247 from Abcam), anti‐Opa1 (1: 1000, #80471 from Cell Signalling), anti‐AMPK*α* (1: 1000, #2532 from Cell Signalling), and anti‐Phospho‐AMPK*α* (Thr172) (1: 1000, #2535 from Cell Signalling). After washing, they proceeded to be incubated with secondary antibodies. Loading controls were VDAC (1: 1000, #4661 from Cell Signalling) and *β*‐actin (1: 1000, #4967 from Cell Signalling). Image J software was used for quantitative analysis.

### 5.9 Enzyme‐Linked Immunosorbent Assay (ELISA)

Interleukin‐1*β* (IL‐1*β*) concentrations were determined using commercial ELISA kits (Abbkine; Cat# KTE7005) according to the manufacturer's instructions. Briefly, mice were anaesthetised and brain tissue was extracted. The tissue was homogenised in 0.9% saline (1 mL per 200 mg tissue) using mechanical grinding. The resulting homogenate was centrifuged at 12,000 rpm for 10 min at 4°C. Standard samples were prepared according to the kit protocol to generate a standard curve. The absorbance of the samples was measured at 450 nm using a microplate reader. Sample concentrations were calculated based on the standard curve.

### 5.10 Luminescent ATP Assay

ATP synthesis in freshly dissected mouse brains was quantified using a Luminescence ATP Detection Assay Kit (Beyotime; S0027) according to the manufacturer's instructions. Brain tissue samples were lysed and adjusted to a concentration of 1 mg/mL. Luminescence was measured using a Varioskan LUX microplate reader (ThermoFisher Scientific). ATP concentrations were determined by normalising the luminescence readings to an ATP standard curve and are expressed as relative fold change.

### 5.11 Real‐Time PCR

Total RNA was extracted using RNAiso Plus protocol (#9108/9109 from Takara). Transcription into complementary DNA was done using PrimeScript RT reagent Kit (#RR047Q from Takara). SYBR Green qPCR assay was performed in the ABI StepOnePlus Real‐Time PCR System (#4376600). Primers sequences are as follows: Mfn‐2, F: CACCGTCAAGAAGGATAA; R: CTGCTCAAAGATTCCATT; Opa‐1, F: GCTATCACTGCCAATACAT; R: TTCTTCTCACCGTCTTCT; Drp‐1, F: TCAGTATCAGTCTCTTCTAA; R: GTTCCTTCAATCGTGTTA; Ppar‐*γ*, F: TGTGGGGATAAAGCATCAGGC; R: CCGGCAGTTAAGATCACACCTAT; Pgc‐1*α*, F: AAACTTGCTAGCGGTCCTCA; R: TGGCTGGTGCCAGTAAGAG; Nrf1, F: GCACCTTTGGAGAATGTGGT; R: GGGTCATTTTGTCCACAGAGA; Nrf2, F: CCAGCTACTCCCAGGTTGC; R: CCTGATGAGGGGCAGTGA; Tfam, F: CCTTCGATTTTCCACAGAACA; R: GCTCACAGCTTCTTTGTATGCTT; COX‐2, F: AACCGCATTGCCTCTGAAT; R: CATGTTCCAGGAGGATGGAG; *β*‐actin, F: CTGAATGGCCCAGGTCTGA; R: CCCTGGCTGCCTCAACAC.

### 5.12 Statistical Analysis

All data were expressed as mean ± SEM. Unpaired student *t*‐tests (two‐tails) were used for comparison between two groups. Kaplan–Meier distribution was used to compare lifespan. *p* < 0.05 was considered statistically significant and *p* < 0.01 considered highly significant.

## Author Contributions

J.L.: conception and design, collection of data, animal experiments, data analysis, and manuscript writing. L.C. and X.X.Z. (Xiaoxian Zhang): conception and design, data analysis. X.X.Z. (Xiaoya Zhou) and L.C.: animal experiments. Q.L.: conception and design, manuscript writing, administrative support, and financial support.

## Ethics Statement

This study was conducted in accordance with the ethical standards of the Institutional Review Board of The University of Hong Kong (HKU) and relevant international guidelines. Animal experiments were performed under protocols approved by the HKU Animal Ethics Committee and adhered to the ARRIVE guidelines. All efforts were made to minimise animal suffering, including the use of humane endpoints and appropriate anaesthesia protocols.

## Conflicts of Interest

The authors declare no conflicts of interest.

## Supporting information


**Data S1:** Supporting Information.

## Data Availability

The data that support the findings of this study are available from the corresponding author upon reasonable request.
