# Supplementary material for: Pioglitazone Ameliorates Mitochondrial Oxidative Stress and Inflammation via AMPK‐Dependent Inhibition of Mitochondrial Fission in Leigh Syndrome
Source: Cell Prolif. 2025 Aug 6;59(3):e70109. doi: 10.1111/cpr.70109 (PMC12961535; doi:10.1111/cpr.70109)
Supplement: Supplementary file 1 — Data S1: Supporting Information. [file CPR-59-e70109-s001.docx]

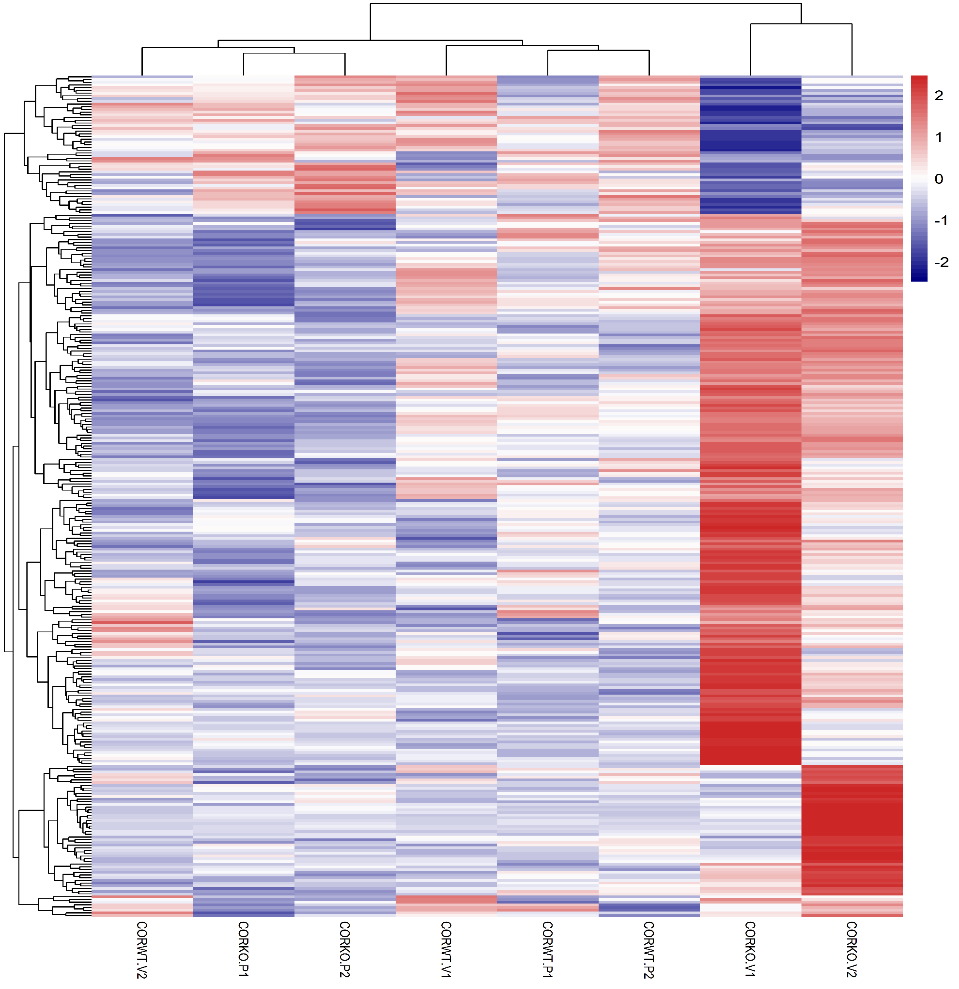
 **A**

**B**


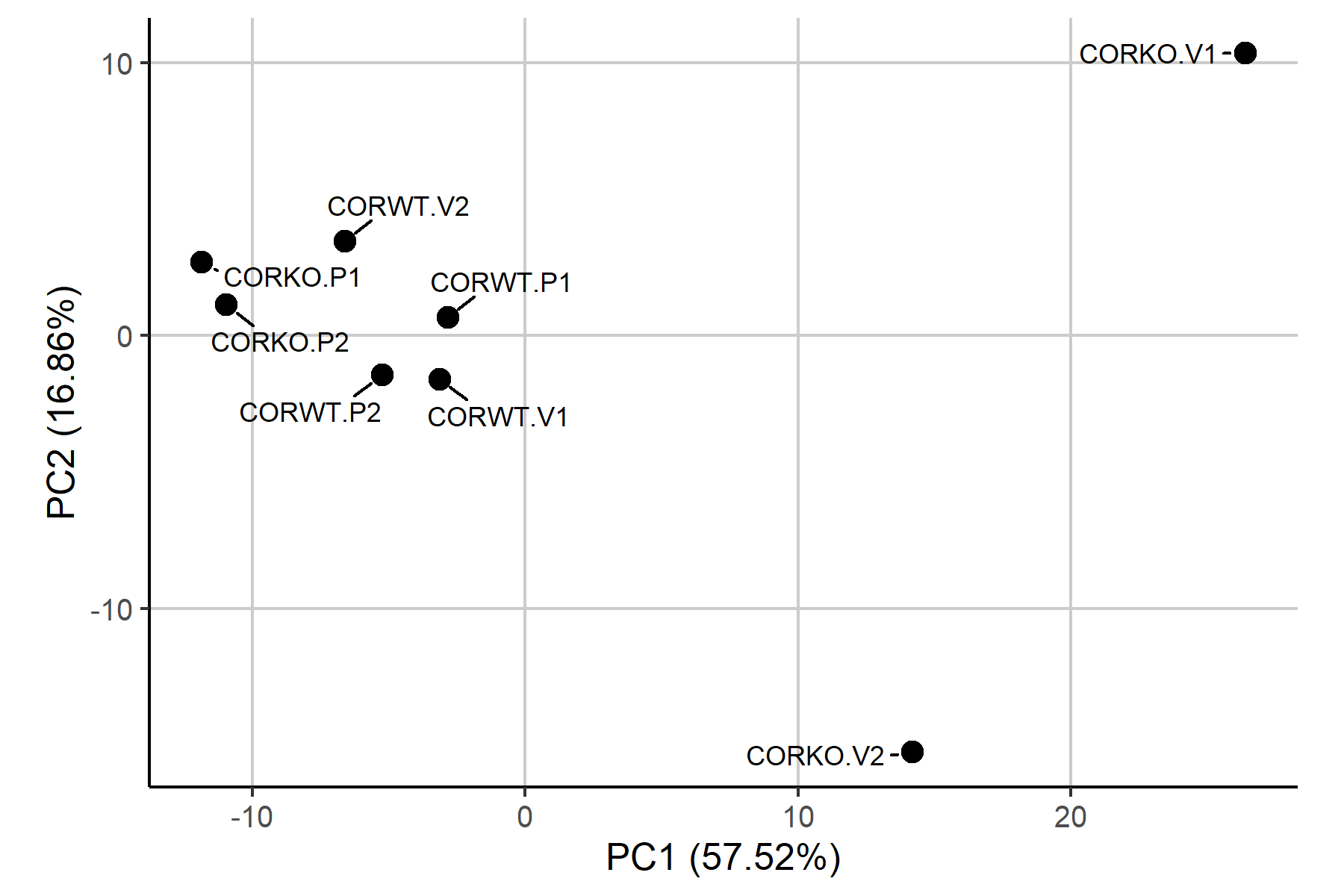


**C D**

**
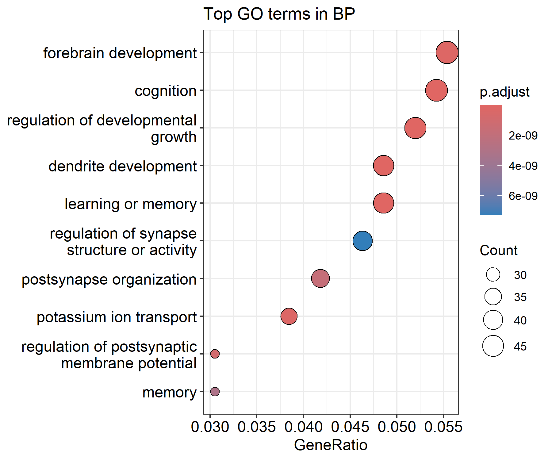
**

**E** **F**


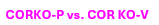

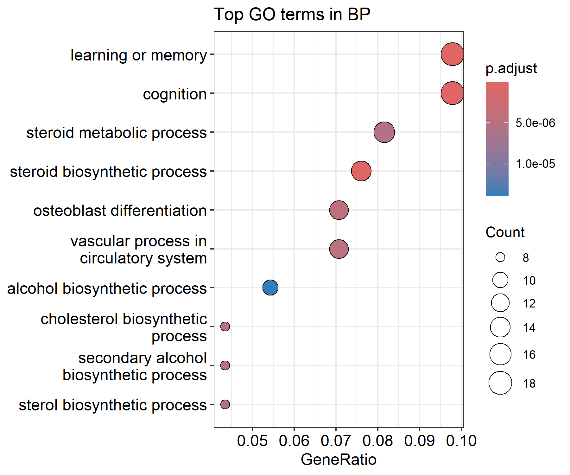


**Figure S1. RNA sequencing of** **cerebral cortex in WT and Ndufs4 KO mice with or without pioglitazone treatment.**

**A**. Heat map depicting the distinct gene expression profile of cerebral cortex in the 4 groups. Colour bar shows gene fold counts normalized across all samples (Padj ≤ 0.05) (n = 2). **B**. PCA depicting gene expression profiles across 8 samples in 4 groups. PC, principal component. **C ,E**. Volcano plot depicting the up-regulated and down-regulated genes. **D**. The GO terms of the BP categories enrichment of the down-regulated genes in cerebral cortex of KO mice relative to WT mice. **F**. The GO terms of the BP categories enrichment of the down-regulated genes in cerebral cortex of KO mice treated with pioglitazone relative to KO mice treated with vehicle. COR, cerebral cortex; V, vehicle treatment; P, pioglitazone treatment.
